# Anæsthetics

**Published:** 1891-11

**Authors:** 


Responses to our invitation, in the last number of the JOURNAL, for communications relative to the treatment of "common colds," one of which appears in this issue, encourage us to continue the feature. For the December JOURNAL we invite letters from our readers on their experience with, and treatment of, ACUTE BRONCHITIS.

### ANÆSTHETICS.

The recent discussion on anæsthetics, by the members of the Atlanta Society of Medicine, disclosed the same diversity of opinion as to the relative safety of ether, chloroform and the A. C. E. mixture, which has characterized similar discussions of the same subject by medical bodies in this country and Europe. Each gentleman preferred the agent with which he was most familiar, and which had given him best results; and while the consensus of opinion was in favor of ether, there was not that unanimity of sentiment in its favor to which its greater relative freedom from danger, in ordinary cases, justly entitles it. There are comparatively few surgeons of large experience, who have given both ether and chloroform a fair trial, who do not give ether the preference. Each is safer than the other in properly selected cases. Both require the undivided attention of an intelligent administrator, who has the capacity for winning the entire confidence of his patient, and explaining to him the disagreeable sensations which these agents produce. Too little stress is laid upon the latter point by text-books and teachers. Surgeons usually select the least reliable assistant to administer the anæsthetic. The poor patient is frequently smothered by a cone or inhaler, without having received preliminary encouragement or instruction, and held down by brute force until the overpowing effects of the drug are obtained. Such an undertaking is often difficult of performance, and not altogether free from danger. Few things are more distressing to witness than the terror-stricken expression, and futile, though almost superhuman struggling of such a victim.

Mixed narcosis has a rather limited field for usefulness. The A. C. E. mixture certainly seems to be an unscientific compound, and of doubtful utility.

We are prone to jump at conclusions from insufficient data. We do not make due allowance for individual susceptibilities and idiosyncrasies. We may hope for an ideal anæsthetic, but in the meantime we must study each patient carefully, and administer to him that one which seems best suited to his particular case. The surgeon who does less is a culpable routinist, who sooner or later finds it out to his sorrow.

